# Voronoi-based analysis of clustering dynamics in experimental volcanic ash clouds

**DOI:** 10.1007/s00445-025-01933-x

**Published:** 2026-01-21

**Authors:** Antonio Capponi, Corrado Cimarelli, Pablo Mininni

**Affiliations:** 1 Department of Earth and Environmental Sciences, Ludwig-Maximilians-Universität München, Theresienstraße 41, 80333 Munich, Germany; 2 Departamento de Física, Facultad de Ciencias Exactas y Naturales, Universidad de Buenos Aires, Ciudad Universitaria, 1428 Buenos Aires, Argentina; 3 Instituto de Física Interdisciplinaria y Aplicada (INFINA), CONICET-Universidad de Buenos Aires, Ciudad Universitaria, 1428 Buenos Aires, Argentina

**Keywords:** Preferential concentrations, Clustering, Volcanic ash, Experimental volcanology, Volcanic clouds, Settling dynamics, Voronoi tessellation

## Abstract

Explosive volcanic eruptions inject large amounts of ash into the atmosphere, where it disperses regionally and globally, posing risks to aviation, infrastructure, and public health. Accurate ash dispersal forecasting is crucial for hazard mitigation, yet current models primarily rely on eruption source parameters, such as particle size distribution, while largely neglecting evolving atmospheric ash distributions. Turbulence-driven particle interactions generate dense clusters that travel faster than isolated particles, shortening the residence time of fine ash and potentially boosting collision and aggregation rates. These processes remain poorly constrained. Here, we present an experimental framework to quantify clustering in controlled ash columns over particle volume fractions *φ* = 10^–5^–10^–2^. Using Laacher See ash (1000–63 µm), we vary particle size distributions and release rates, acquire high-speed laser-illuminated videos for particle tracking, and apply Voronoi tessellation to quantify preferential concentration. We find that particle-driven convection intensifies with decreasing size, while varying *φ* modulates clustering across all sizes < 500 µm. Clustering produces strongly inhomogeneous distributions within the column, enhances particle–particle interactions, and likely promotes aggregation. It also affects settling, as smaller particles within clusters can settle faster than larger, unclustered ones, thus challenging traditional assumptions that link particle size to settling velocity. Incorporating these dynamics into dispersal models, and accounting for their signatures in remote-sensing retrievals, should improve forecast accuracy and refine our understanding of volcanic ash transport and deposition.

**Supplementary information:**

The online version contains supplementary material available at 10.1007/s00445-025-01933-x.

## Introduction

Volcanic plumes are complex mixtures of volcanic gases, ash particles, and entrained air injected into the atmosphere during explosive eruptions. While larger particles typically settle near the source, fine-grained ash (< 100 µm) can be transported over vast distances both regionally and globally. Cloud dispersal mostly depends on atmospheric conditions (e.g. wind, temperature, and humidity) as well as particle-gas-air interactions (Sparks et al. 1997). Volcanic ash poses significant risks to aviation, including damaging instrumentation and causing engine failure (Kueppers et al. 2014). Beyond aviation, ash impacts infrastructure, contaminates water supplies, and threatens human health (Durant et al. 2010). Accurate detection of ash and forecasting of cloud evolution are therefore crucial for aviation safety, risk assessment, and emergency response planning. Volcanic ash transport and dispersion models (VATDMs) are widely used to forecast the distribution of volcanic ash in the atmosphere (e.g. Jones et al. 2007; Folch et al. 2010; Folch 2012; Beckett et al. 2020, 2022). However, these models are often subject to errors, primarily due to inaccuracies in parameterizing eruption source parameters such as ash particle size distribution (PSD) and concentration (e.g. Folch 2012; Francis et al. 2012; Dacre et al. 2016; Capponi et al. 2022a). To improve accuracy, VATDMs are frequently coupled with remote sensing data, which helps forecasting regions of hazardous ash loadings. Despite this integration, uncertainties persist, arising from models’ inability to fully capture the complex physical processes within volcanic clouds and limitations in accurately characterizing eruption source parameters (e.g. Folch 2012; Bonadonna et al. 2012; Egan et al. 2020). Ground-based remote sensing instruments offer potential for deriving parameters such as particle concentration and size distribution, yet their application is limited because, without a deep understanding of particle settling dynamics and those parameters influencing them, remote sensing data cannot be accurately interpreted (Marzano et al. 2013).

The development of particle inhomogeneous distributions (i.e. preferential concentrations) in a volcanic cloud is one of those processes that are not fully grasped by current models and are still difficult to derive from remote sensing data. Inhomogeneous distributions arise from the formation of clusters, i.e. regions where particles are densely concentrated and surrounded by areas of lower particle density (‘voids’). The formation of these clusters has significant implications for particle settling behaviour. Dense clusters settle at higher velocities than the surrounding dilute regions, potentially through settling-driven gravitational instabilities within the cloud (e.g. Manzella et al. 2015; Scollo et al. 2017). Furthermore, clustering can promote ash aggregation, as densely packed particles are more likely to collide and coalesce into larger aggregates, further accelerating their premature removal from the volcanic cloud (Lane et al. 1993). Failing to detect and account for these processes can lead to inaccurate forecasts of ashfall distribution and deposition, contributing to the underestimation of ashfall in proximal areas and overestimation of concentrations in the distal cloud (Taddeucci et al. 2011; Brown et al. 2012; Folch 2012). To mitigate the impact of volcanic ash, it is therefore critical to gain a deeper understanding of the mechanisms triggering the development of inhomogeneous distributions within volcanic clouds, their controlling parameters, and their influence on ash settling behaviour.

Clustering, where particles concentrate in specific regions of a flow, is a well-established phenomenon in fluid mechanics (e.g. Squires and Eaton 1991; Eaton and Fessler 1994; Saw et al. 2008), particularly within turbulent flows, and plays a key role in processes like dispersion of pollutants in the atmosphere, cloud formation, and combustion. On the one hand, numerical and experimental studies have shown that spherical particles with small inertia tend to accumulate in regions of low vorticity, such as regions of flow convergence, due to interactions between particle inertia and the surrounding flow structures (Eaton and Fessler 1994). On the other hand, spherical particles with large inertia are expelled from the eddies and are believed to accumulate in points with low Lagrangian acceleration of the fluid, i.e. in regions in which forces are small in the reference frame of the fluid (Goto and Vassilicos 2008; Obligado et al. 2014). The level of clustering depends on the Stokes number (St), which measures the particle’s response time to the flow. Clustering is strongest when St is around 1, as the particle response time matches the smallest turbulent timescales (Brandt and Coletti 2022). At large St values, particle inertia dominates, causing them to move independently of the flow. By contrast, at very small St, particles align with high-strain, low-rotation regions of the flow (Voth and Soldati 2017). Gravity further influences clustering, especially for heavy particles, causing vertically elongated clusters, as observed in both simulations and experiments. These dynamics become more complex when considering nonspherical, anisotropic particles, such as ellipsoids and fibres, whose shape and inertia significantly affect their behaviour in turbulent flows. For instance, small fibres align with flow vorticity, while disks tumble more than they spin (Voth and Soldati 2017).

Insights from fluid mechanics provide a solid base for investigating particle clustering within volcanic clouds and pyroclastic density currents (PDCs). Recent numerical simulations have linked turbulence-induced particle clustering to the formation of lightning rings in volcanic plumes, highlighting the significant role of multiphase turbulent flow dynamics in such phenomena. The simulations showed that heavy particles concentrate in regions of high turbulence intensity, such as the updraft and umbrella cloud, driving particle collisions and enhancing the likelihood of charge separation (Ichihara et al. 2023), a key process in lightning generation (Cimarelli et al. 2014; Cimarelli and Genareau 2022). In PDCs, gas–particle interactions are now recognized as key controls on flow behaviour. Multiphase models and experiments show that St-dependent segregation, particle clusters, and self-generated turbulence strongly influence the internal structure, runout, and impact of PDCs (e.g. Burgisser and Bergantz 2002; Breard et al. 2016; Breard and Lube 2017; Lube et al. 2020; Brosch et al. 2022; Uhle et al. 2024). These studies demonstrate that particle-laden gravity currents can self-organize into dense, clustered regions embedded in coherent turbulent structures, with gas–particle and wake interactions enhancing mobility and focusing destructive dynamic pressures. However, PDC work has focused mainly on near-surface gravity currents; how similar interactions operate in dilute, gravitationally settling ash clouds remains less well constrained. Experimentally, recent investigations using water-particle suspensions in density-stratified tanks have provided new insights on how sedimentation-driven gravitational instabilities (SDGIs) and the formation of particle-laden fingers influence particle settling in volcanic ash clouds (Manzella et al. 2015; Scollo et al. 2017; Fries et al. 2024). These processes are controlled by particle size, volume fraction (*φ*), and flow coupling, with finer particles and higher *φ* favouring finger formation. The number and speed of fingers increase with particle concentration and are independent of particle composition, emphasizing the dominance of physical processes over chemical properties in finger formation. Clustering and gravitational instabilities become pronounced once *φ* enters the moderate (two-way) coupling regime (∼10^–6^–10^–3^; Elghobashi 1994), with natural volcanic eruptions exhibiting *φ* at neutral buoyancy of ∼10^–6^–10^–5^ and boundary-layer *φ* up to ∼10^–4^, and laboratory analogues spanning *φ* of ∼7 × 10^–4^–7 × 10^–3^ and boundary-layer *φ* of ∼10^–3^–10^–1^ (Carazzo and Jellinek 2012; Fries et al. 2024). Depending on plume heights, field-scale inversions estimated *φ* of ∼10^–5^–10^–6^ at distal regions and of ∼10^–3^ proximally (Bursik et al. 1994; Del Bello et al. 2017). Experimentally, gravitational instabilities were observed only for particles < 125 μm (Carazzo and Jellinek 2012) and for St < 1, highlighting the critical role of fine ash in driving these dynamics. Fingering instabilities consistently initiate once *φ* exceeds ∼10^–6^ and intensify through two-way coupling toward strong coupling (the so-called four-way coupling in which particles not only affect the flow but also interact between themselves; Elghobashi 1994) above ∼10^–3^ (Fries et al. 2024; Foster et al. 2025). Inside the fingers, divergence and vorticity analyses indicate heterogeneity in particle concentration and increased turbulence, also possibly promoting particle aggregation at cloud bases and within fingers (Manzella et al. 2015; Scollo et al. 2017; Fries et al. 2024).

While studies on finger formation in density-stratified fluids (e.g. Carazzo and Jellinek 2012; Manzella et al. 2015; Scollo et al. 2017; Fries et al. 2024) provide valuable insights into SDGIs and particle segregation, these experiments primarily focus on buoyancy-driven instabilities. As such, they may not fully capture the complex turbulent processes and particle-particle interactions that might occur within volcanic clouds. To address this gap, here we present a novel experimental framework and accompanying data analysis approach developed to specifically investigate the formation and evolution of particle clusters within a controlled environment. Similarly to Del Bello et al. (2017), we focus on experimental falling columns of natural volcanic ash in which particle-induced perturbations of the carrier fluid (two-way coupling) and particle-particle interactions (four-way coupling) are negligible below *φ* ≈ 10^–6^ but become increasingly important up to *φ* ≈ 10^–3^ (Del Bello et al. 2017). More specifically, we examine how these coupled mechanisms shape the onset and evolution of particle clustering. Our newly developed apparatus allows the generation of sustained free‐falling particle columns inside a closed chamber, with *φ* ranging between 10^–5^ < *φ* < 10^–2^. As ash falls, it entrains ambient air and, upon impact with the floor, drives a sustained recirculation loop and localized turbulence. This self-generated, settling-driven turbulence continuously redistributes particles, which in turn alter the flow field. By varying particle size distribution and volume fraction, we investigate how these coupled fluid–particle processes control the development of clustering. Furthermore, we apply Voronoi tessellation to quantify preferential concentration, a technique only recently adopted for particle‐laden turbulent flows (Monchaux et al. 2010). In this framework, each particle Voronoi cell area is inversely proportional to its local concentration, allowing precise discrimination between clustered and dispersed particles. By combining our closed-chamber experiments with Voronoi analysis, we not only reveal new details of clustering dynamics in experimental volcanic ash columns. We also establish a quantitative framework for assessing how turbulence and volume fraction govern particle segregation at the smallest scales, introducing a methodology that can be used in other laboratory configurations –  for example, where turbulence is excited using other mechanisms or where fluids stratified in density are considered.

## Methods

### Experimental apparatus

**Fig. 1:**
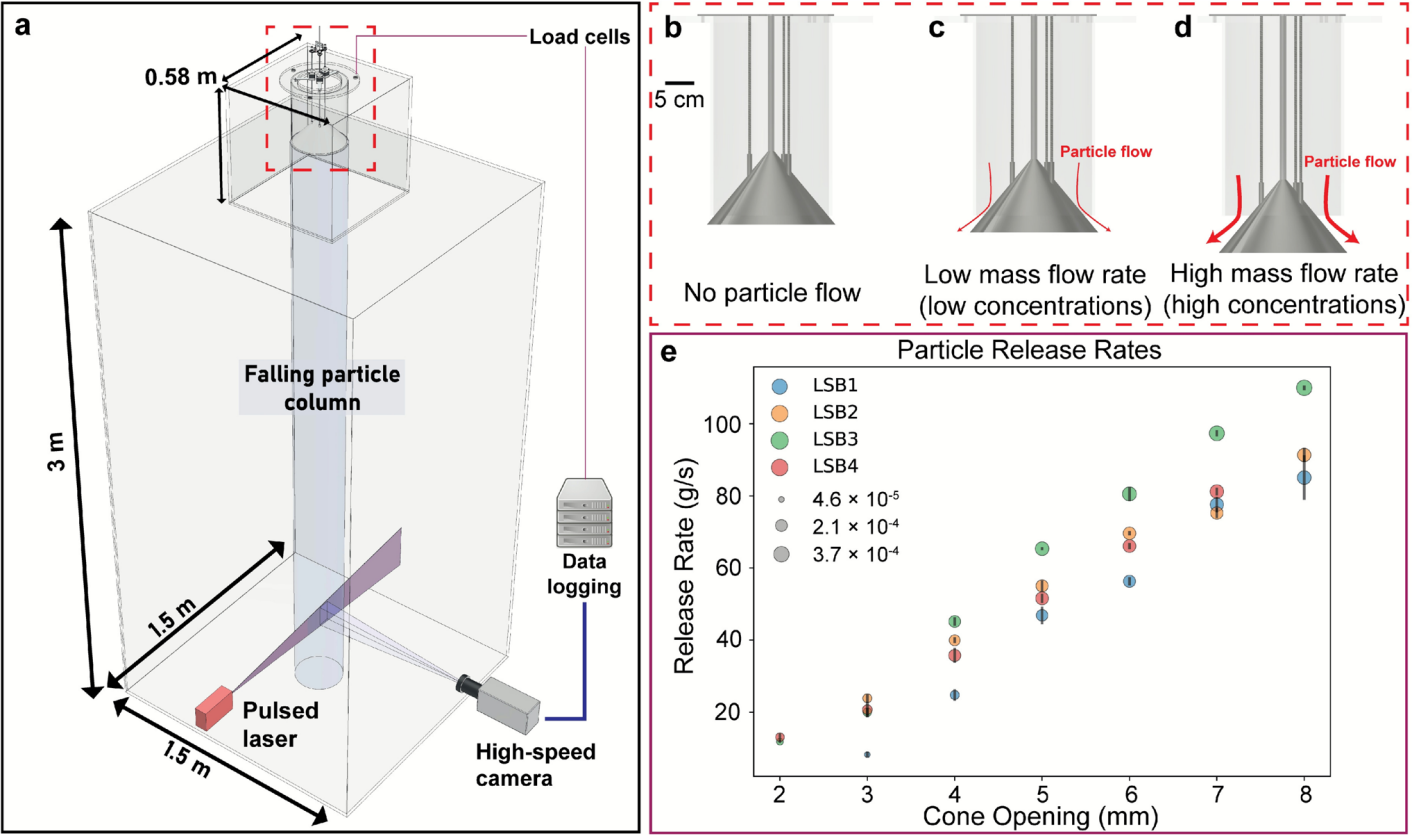
**a** Experimental apparatus consisting of the release system and a large settling chamber, equipped with a particle image velocimetry system. **b**–**d** Illustrations of the cone in different positions: **b** fully closed and sealing the hopper, **c** partially open for low particle release rates, and **d** further open for high particle release rates. **e** Range of particle release rates (g/s) as a function of cone opening (mm) and particle sized distributions (represented by different colours). Each data point represents the mean release rate from at least three experimental runs under identical conditions. Most of the error bars are smaller than the symbols, and as a result may not be clearly visible. Symbol sizes in **e** represent the bulk particle volume fraction (*φ*_*b*_) calculated from the measured release rates, the mean terminal fall velocities (Table 1), and for a column diameter of 27 cm. Note that these *φ*_*b*_ represent estimates only, as they assume a uniform distribution within the column, which is not the case in these experiments

We conducted the experiments in a newly developed experimental fall chamber, building on the apparatus described in Capponi et al. (2022b). The apparatus (Fig. 1a) consists of a particle dispersion system (0.58 × 0.58 × 0.58 m) located above a large particle sedimentation chamber (1.5 × 1.5 × 3 m).

The dispersion system is designed to generate sustained and repeatable columns of falling particles for durations of up to several minutes. At its core is a custom-designed conical valve that ensures uniform mass flow discharge of particulates, such as volcanic ash. The system includes a cylindrical hopper (30 × 20 cm) for material storage, sealed by a cone. Lowering the cone creates an annular gap, allowing for a controlled and adjustable mass flow of particles. The release rate increases proportionally with the downward movement of the cone (Fig. 1b-–d), enabling controlled particle discharge. The cone movement is precisely controlled by three high-resolution linear actuators (Nanotec LA281S10-A-UGFC; 0.025 mm/step, max speed 120 mm/s), ensuring repeatable and accurate release. The precise control over the release rate enables the creation of falling columns with varying particle concentrations, all while maintaining repeatability across experiments. Fast-response load cells (Kern load cells CK30-Y1) support the entire release system, continuously monitoring the release rate to ensure precise mass flow measurements and reliable particle volume concentration estimates (Fig. 1e). Once the hopper is filled, the particles remain undisturbed until the release process starts. As the cone is lowered from its sealed position, it opens the annular gap, allowing for uniform particle discharge, ensuring that particles within the same cross-section are released together. This minimizes segregation and ensures consistent flow. To further assist the flow and prevent blockages, the hopper is equipped with vibration motors. These motors are only activated as needed (i.e. for large PSDs released at small cone opening) just at the onset of discharge, to facilitate smooth material release, working in conjunction with the cone movements and at low amplitude. As soon as the particles begin flowing, gravitational forces dominate over the mild vibration, preventing any size-dependent sorting. High-speed imaging throughout the run consistently shows a polymodal particle distribution, with no evidence of vertical grading.

The free-fall of particles occurs within the main draught-proof settling chamber. Two sides of the chamber are fitted with large (1.5 × 3 m) optically transparent panels, while the remaining sides are constructed from dark, opaque materials. One of the opaque sides features a 1.8 × 0.8 m door, providing easy access to the chamber after each experiment. The optically transparent panels serve as viewing windows and are needed for imaging the experiments using a particle image velocimetry (PIV) system. The PIV system consists of an Oxford Laser Firefly 300 W pulsed laser (808 nm, 0–48 kHz pulse rate) and a Phantom V711 high-speed camera, positioned at 90° to the laser. Both the laser and the camera are mounted on rails, enabling movement along the entire height and length of the chamber, facilitating measurements at various locations. The camera, equipped with a Zeiss 100 mm 2.8 macro-lens, acquired 10 bits images of the falling particles at 5000 frames per second (fps) and 8 µm pixel resolution. The imaged volume is a region of 127 × 64 × 4 mm, with a laser sheet thickness of 2 mm, from a working distance of 810 mm. The imaged area is located 2.5 m below the release system.

Experiments are conducted at ambient pressure, with a temperature of 22–24 ± 0.45 °C and relative humidity of 50–60% ± 4.5% RH. These conditions are continuously monitored by a Sensirion SEN55 environmental sensor node, installed within the main settling chamber.

### Starting material

For our experiments, we used four different PSDs of the well-characterized (Douillet et al. 2014) phonolitic ash from Laacher See (East Eifel volcanic field, Germany), sieved into the following classes (Table 1): 1–0.5 mm (LSB1), 0.5–0.25 mm (LSB2), 0.3–0.125 mm (LSB3), and 0.125–0.063 mm (LSB4). Good sorting within the considered ranges was confirmed by capturing images of loose particles using a scanning electron microscope (SEM). These images were then analyzed using a custom Python code developed for automated shape analysis. The code identifies the contours of each particle and calculates key parameters such as area, minor and major axes, Feret diameter, perimeter, axial ratio, circularity, concavity, form factor, solidity, and convexity. For all the experiments, we released the same mass of particles, 600 g, across a range of different cone openings, to achieve different constant release rates (Fig. 1e; Table 1). To ensure reproducibility of the ash releases and their measurements, each release was repeated at least three times. As we are interested in exploring the influence of particle size and volume fractions on the development of clusters within the falling column, we consider the Stokes number, which serves as a proxy for particle size and characterizes the behaviour of particles suspended in a fluid flow, approximated as

$$St={~}^{{\rho }_{p}{D}^{2}{U}_{p}}\!\left/ \!{~}_{18{\mu }_{a}L}\right.$$

where *ρ*_*p*_ is the particle density, for which we use a single value of 1364 kg/m^3^, representing the mean pumice density reported by Douillet et al. (2014) for the Laacher See deposit. Across the 0.125–2.5 mm size range, the reported densities of the individual grain-size fractions differ from this mean by only a few percent (Douillet et al. 2014), so this choice does not introduce any meaningful scaling of the Stokes number between PSDs. *µ*_*a*_ is the air dynamic viscosity (1.8 × 10^−5^ Pa s at 25 °C), and *D* is the mean particle diameter of each PSD. *L* is taken as half the chamber width, 0.75 m, as in a confined column the largest buoyancy-driven vortices cannot exceed half the span between the walls, and *U*_*p*_ is the root-mean-square fluid velocity at scale *L*. *U*_*p*_ was estimated from the particle trajectories (obtained using particle tracking velocimetry) as the root mean square of the horizontal and vertical particle-velocity fluctuations, $U_{\mathit p}\mathit=\sqrt{\mathit{\left({u_{p,x}^{'2}+\left\langle u_{p,z}^{'2}\right\rangle}\right)}}$, where $u_{\mathit p\mathit,\mathit x}^{\mathit'\mathit\;}\mathit\;$ and $u_{p\mathit,z}^{\mathit'\mathit\;}\mathit\;$ are the fluctuating particle-velocity components obtained from one-frame displacements after filtering out short or noisy tracks, removing large frame jumps, and subtracting the frame-wise median settling velocity.

**Table 1:** Summary of experimental parameters and release characteristics

*PSD (mm)*	*Cone opening (mm)*	*Release rate (g/s)*	*Bulk concentrations C*_*b*_* (kg/m*^*3*^*)*^*a*^	*Bulk volume fraction (φ*_*b*_*)*	*φ*^*b*^	*St*	*Mean u*_*p*_*(m/s)*
*LSB1 (1–0.5)*	3–8	8.2–85	6.3 × 10^–2^ – 3.7 × 10^–1^	4.6 × 10^−5^–2.7 × 10^−4^	8.9 × 10^–4^ – 1.5 × 10^–2^	1.20–1.51	2.27–4
*LSB2 (0.5–0.25)*	3–8	24–91	1.6–3.5 × 10^–1^	1.2 × 10^−4^–2.6 × 10^−4^	10 × 10^–4^ – 1.3 × 10^–3^	0.34–0.36	2.6–4.5
*LSB3 (0.3–0.125)*	2–8	10–110	8.5 × 10^–2^ – 5 × 10^–1^	6.2 × 10^−5^–3.7 × 10^−4^	1.4–6.9 × 10^–4^	0.14–0.29	2–4
*LSB4 (0.125–0.063)*	2–7	8.3–81	1.2–3.7 × 10^–1^	8.8 × 10^−5^–2.7 × 10^−4^	1.4–3.2 × 10^–5^	0.02–0.07	1.1–3.8

^a^Bulk concentrations estimated from the logged release rates, the mean terminal fall velocities (*u*_*p*_) and for a column diameter of 27 cm

^b^Bulk volume fractions *φ*_*b*_ are obtained from bulk concentrations *C*_*b*_, considering *ρ*_*p*_ = 1364 kg/m^3^

^c^Calculated by converting Voronoi cell areas into volume fractions, as described in the “Data processing” section

### Data processing

The camera frame rate provides a high temporal resolution, with 5000 frames (spanning a time window of 1 s) saved for each experiment. This frame count is enough to ensure that transient dynamics and subtle temporal fluctuations are accurately resolved, offering a robust dataset for both instantaneous and time-averaged flow characterization. The large frame count covers multiple flow cycles, effectively capturing a wide range of particle interactions and providing a comprehensive representation of the flow’s variability and steady-state behaviour. Using the same characteristic *L* as in the Stokes number definition and column velocities of 1.1–4.5 m/s (Table 1), the associated large-eddy turnover time would be of order 0.2–0.7 s. Each 1 s sequence therefore spans roughly 1.5–6 turnover times of the most energetic motions. For runs conducted under the same experimental conditions, snapshots of the flow were taken at key stages of the release—either at its midpoint or at the start, midpoint, and end. This approach not only facilitates a thorough temporal comparison but also ensures comprehensive analysis and reproducibility by accounting for potential variations in flow structure over the duration of the release.

**Fig. 2:**
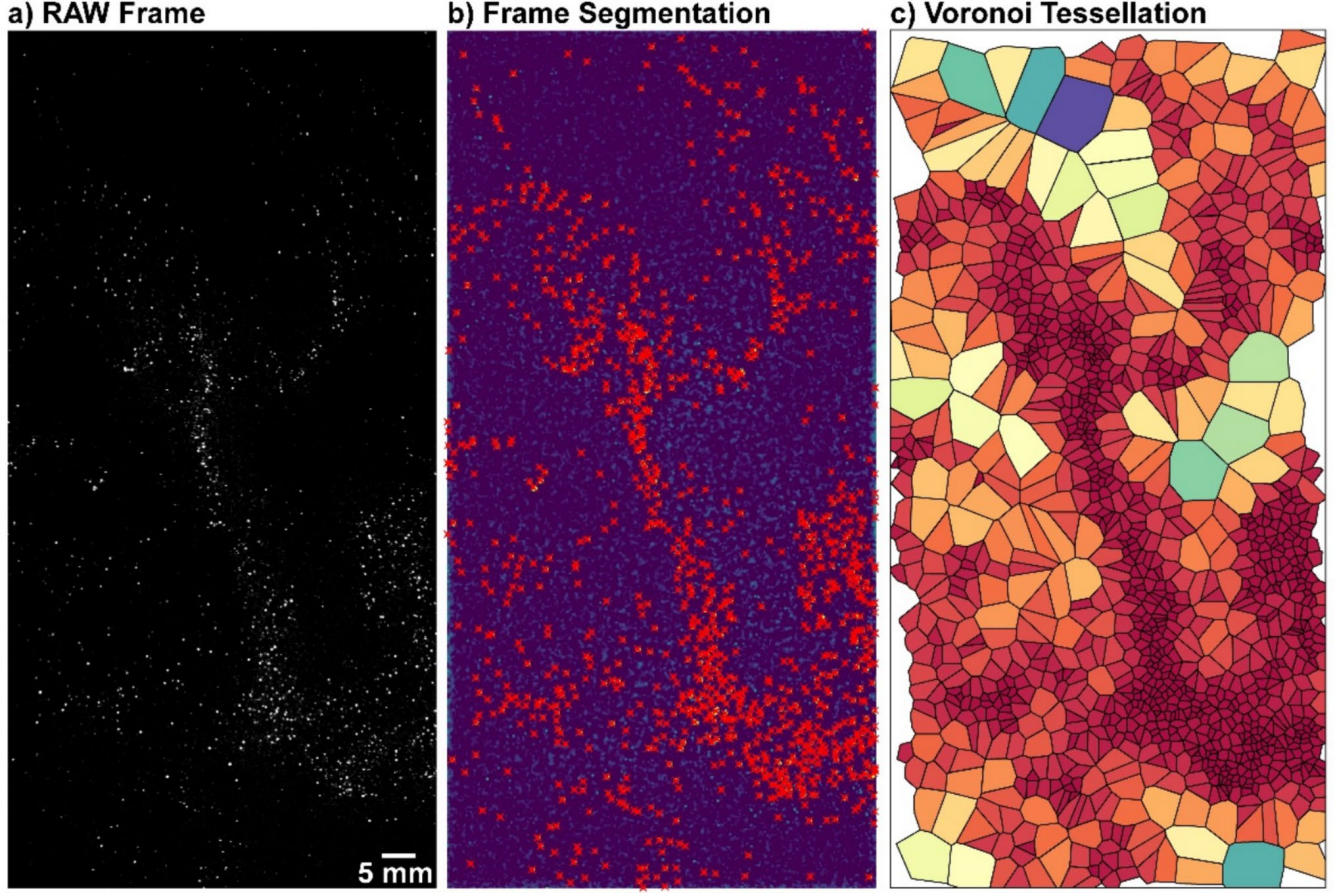
Example of **a** a typical RAW image for LSB3 before post-processing, **b** after segmentation and particle identification, and **c** the associated Voronoi diagram. Basic levels and contrast adjustments were applied to the frame in **a** for improving visibility

To investigate and quantify particle distribution within the column, we used Voronoi tessellation. Originally introduced as a tool for studying granular systems, Voronoi tessellation has recently been applied to investigate particle behaviour in turbulence (Monchaux et al. 2010, 2012) and is being increasingly used in fluid mechanics for analyzing clustering in particle-laden flows in both experimental and numerical studies (e.g. Obligado et al. 2011, 2014; Marchioli 2017; Mora et al. 2021; Wang et al. 2024). Additionally, the computational efficiency of generating Voronoi diagrams makes them an ideal tool for analyzing spatial distributions in such flows. This method uniquely partitions two-dimensional space into distinct cells, each corresponding to an individual inertial particle. The Voronoi cell associated with a particle encompasses all points in the domain that are closer to it than to any other particle. Figure 2a shows a frame from one of our experiments and how from each identified particle (Fig. 2b) we can then create a Voronoi diagram (Fig. 2c). From its definition, the area, *A*, of each Voronoi cell is the inverse of the local 2D-concentration of particles.

To generate a Voronoi diagram, the first step is to identify each particle within the images. We do so by using MyPTV, an open-source Python algorithm specifically designed for 2D and 3D particle tracking velocimetry (Shnapp 2022). For each experiment, we used MyPTV to perform segmentation across all available images (Fig. 2a), extracting the x-y coordinates of particles. These particles were identified as local intensity maxima exceeding a pre-selected threshold (Fig. 2b). Furthermore, by using the MyPTV tracking function, it is possible to track the particles’ positions over time, thus obtaining, for each particle, its trajectory, velocity, and acceleration.

Then, using Python’s Freud library (Ramasubramani et al. 2020), we generated a Voronoi diagram for each set of particle coordinates at each acquisition time step (Fig. 2c). Due to the finite size of the camera field of view, some cells at the diagram’s border have infinite areas. To ensure the accuracy of our analysis, we excluded these border cells and retained only those particles whose Voronoi cells were fully contained within the visualization window, thereby eliminating potential edge effects and maintaining a consistent dataset for further analysis. For a study of the impact of boundaries in Voronoi tessellation of experimental data, and for comparisons between different methods to alleviate their effects, see, e.g. Angriman et al. (2022).

When investigating the tendency of particles to develop clusters and voids (i.e. regions of higher and lower concentrations), we compared the observed particle distribution to a random Poisson process (RPP; Monchaux et al. 2010). Note that an RPP is the distribution expected for the areas of the Voronoi cells if the particles are homogeneously distributed. Thus, we quantified clustering by analyzing the probability density function (PDF) of the normalized Voronoi cell areas, $V=A/\langle A\rangle$, measured for each experiment, and by quantifying its deviations from the expected PDF for an RPP (e.g. Monchaux et al. 2010, 2012; Obligado et al. 2011, 2014). An excess of probability of finding small Voronoi cells or very large Voronoi cells, compared with the probability predicted by the RPP, indicates the existence of clusters or voids, respectively. Furthermore, normalizing each cell area, *A*, by the average area, $\langle A\rangle$, allows for the comparison of different experimental conditions independent of variations in particle volume fractions, as the normalization ensures a mean value of 1. This approach focuses on the distribution shape and spread, which directly reflects the degree of clustering. For the PDF analysis, we used two parallel approaches to ensure robust and unbiased results. First, we calculated the PDFs using all frames from each experiment, with frames randomly selected to avoid potential temporal correlations. Then, to assess the consistency and evaluate dispersion within subsets, we divided the frame dataset of each experiment into 10 subsets, each comprising 500 randomly selected frames (RSF). The PDFs were then computed independently for each subset. We did not observe any significant divergence among the subset PDFs, indicating strong statistical consistency. Furthermore, the results demonstrated that good statistical convergence could be achieved using a single subset of 500 RSF.

For an RPP, 2D normalized Voronoi areas have a variance of 1.280, corresponding to a standard deviation *σ*_*V*_ ≈ 0.53. While no exact analytical solution exists for the full PDF, approximations often involve fitting it to Gamma distributions (Monchaux et al. 2010; Obligado et al. 2011, 2014; Marchioli 2017). Here, we adopt Ferenc and Néda (2007) analytical model as our RPP reference:

$${PDF}_{2D}\left(y\right)= \frac{343}{15}\sqrt{\frac{7}{2\pi }}{y}^{5/2}exp\left(-\frac{7}{2}V\right)$$

where *V* is the normalized area using the mean area, i.e. $V=A/\langle A\rangle$. Note that the comparison of the observed statistics with the RPP allows us to discriminate the sporadic accumulation of particles that can take place in any random spatial distribution of points in space, from the stronger and dynamically induced clustering of particles caused by the particles’ inertia and by the interaction of the particles with the generated turbulence.

**Fig. 3:**
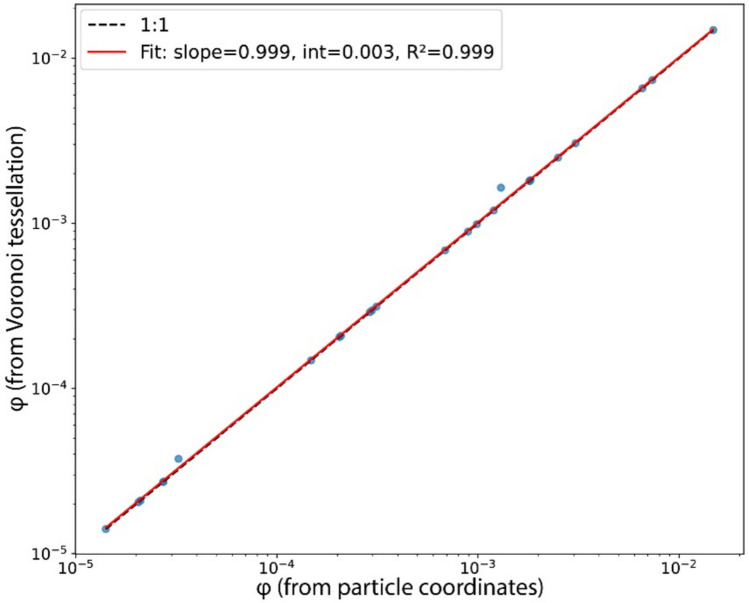
Comparison of particle volume fractions (*φ*) measured via direct measurement from detected particles (x axis) versus Voronoi tessellation (y axis). Blue markers show individual experiments, the black dashed line is the 1:1 reference, and the red line is a linear regression (slope = 0.999, intercept = 0.003, *R*^2^ = 0.999). The near-unity slope, near-zero intercept, and high *R*^2^ confirm that the Voronoi‐based method reproduces true *φ* without systematic bias over the whole range of particle volume fraction, providing also a calibration for the analysis

Finally, particle concentrations and volume fractions, *φ,* in the falling column were determined using different approaches. First, we estimated concentration from the logged release rates. Released particles flow through a constant ~ 0.057 m^2^ cross‐sectional area (assuming a 27 cm diameter column) at rates of ~ 8–110 g/s and mean velocities of ~ 1.1–4.5 m/s, yielding bulk concentrations (*C*_*b*_) on the order of 10^–2^–10^–1^ kg/m^3^ (Table 1). With particle density *ρ*_*p*_ = 1364 kg/m^3^, we also express this as a bulk particle volume fraction *φ*_*b*_ = *C*_*b*_*/ρ*_*p*_ (Table 1, Fig. 1e), which provides a column-averaged lower bound because it assumes a uniform cross-sectional distribution and a fixed column diameter. Second, we calculated *φ* directly from each video frame using two image‐based methods. Both methods produced closely matching *φ* over nearly three orders of magnitude (10^–5^–10^–2^; Fig. 3). In the first method, we identify particle centroids in each frame, assign each a volume from the experiment-specific size distribution, sum the per-frame solid volume, and divide by the imaged control volume to obtain *φ*. In the second, we compute the inverse of each Voronoi cell area to obtain a local 2D particle number density. Dividing by the camera depth of field yields a 3D number density, which, multiplied by the effective particle mass (from the experiment-specific PSD), gives the local mass concentration. The per-frame particle volume fraction *φ* is then obtained by summing these local masses over the frame and dividing by the imaged volume. Figure 3 demonstrates the very close agreement between these two methods: plotting the volume fraction from Voronoi tessellation against that from direct particle‐coordinate summation shows all points falling along a 1:1 line over almost three orders of magnitude (10^–5^–10^–2^). This alignment confirms that, under our imaging conditions, the Voronoi‐based calculation neither under- nor over-estimates the particle volume fraction.

Although this validation shows that *φ* can be measured reliably, converting cell areas into mass or volume fractions requires additional assumptions: a particle size distribution for each centroid (as we are unable to directly run shape analysis from our images) and perfectly uniform illumination across the field of view. Each assumption introduces its own uncertainty. By contrast, Voronoi cell areas provide a pure, geometry-based measure of local number-density fluctuations without propagating uncertainties from particle size or imaging depth. Also, it is the only metric in Voronoi tessellation for which an exact RPP benchmark exists (*σ*_*V*_ ≈ 0.53). Therefore, in line with standard practice in Voronoi tessellation studies (e.g. Feng et al. 2024; Ferenc and Néda 2007; Marchioli 2017; Monchaux et al. 2010, 2012; Mora et al. 2021; Obligado et al. 2011, 2014; Wang et al. 2024), we base our clustering analysis on the normalized cell‐area distribution $A/\langle A\rangle$. Converting to *φ* remains straightforward, but anchoring our clustering metric to $A/\langle A\rangle$ avoids introducing size-, depth-, or illumination-related errors and facilitates direct comparison with prior and future work, even in cases with different particle loads or under different flow conditions.

## Results

In all our experiments, the ash flow through the release system was well controlled and repeatable, with a complete discharge of the ash from the hopper in each run. Shortly after the release had started, the ash formed a well-constrained column of free-falling particles within the settling chamber, with a diameter of 24–30 cm depending on PSDs and release rates. The dynamics of the falling particle column varied significantly depending on particle size and release rate.

Coarser particles (LSB1) showed the most stable behaviour, settling uniformly across most experimental conditions, regardless of release rate (Supplementary Video 01–02). For both LSB2 and LSB3, while still exhibiting some degree of column stability, we observed a similar shift in particle distribution with increasing *φ*. Compared to LSB1, at lower *φ*, smaller, isolated dense regions were more evident (Supplementary Video 03, 05). However, higher *φ* led to larger, more coherent dense regions of particles, sometimes occupying most of the recorded frames (Supplementary Video 04, 06).

Finer particles (LSB4) were characterized by the most dynamic behaviour. Despite the formation of an approximate 25 cm diameter core column, particle-air interactions were pronounced at the edges, especially at slower release rates which also exhibited extended-release durations. For LSB4, we observed distinct clustering with visible and rapidly evolving regions of dense accumulation and sparse voids at lower *φ* (Supplementary Video 07). As *φ* increased, clustering persisted, but the patterns became less distinct, suggesting an increased influence of particle interactions (Supplementary Video 08).

**Fig. 4:**
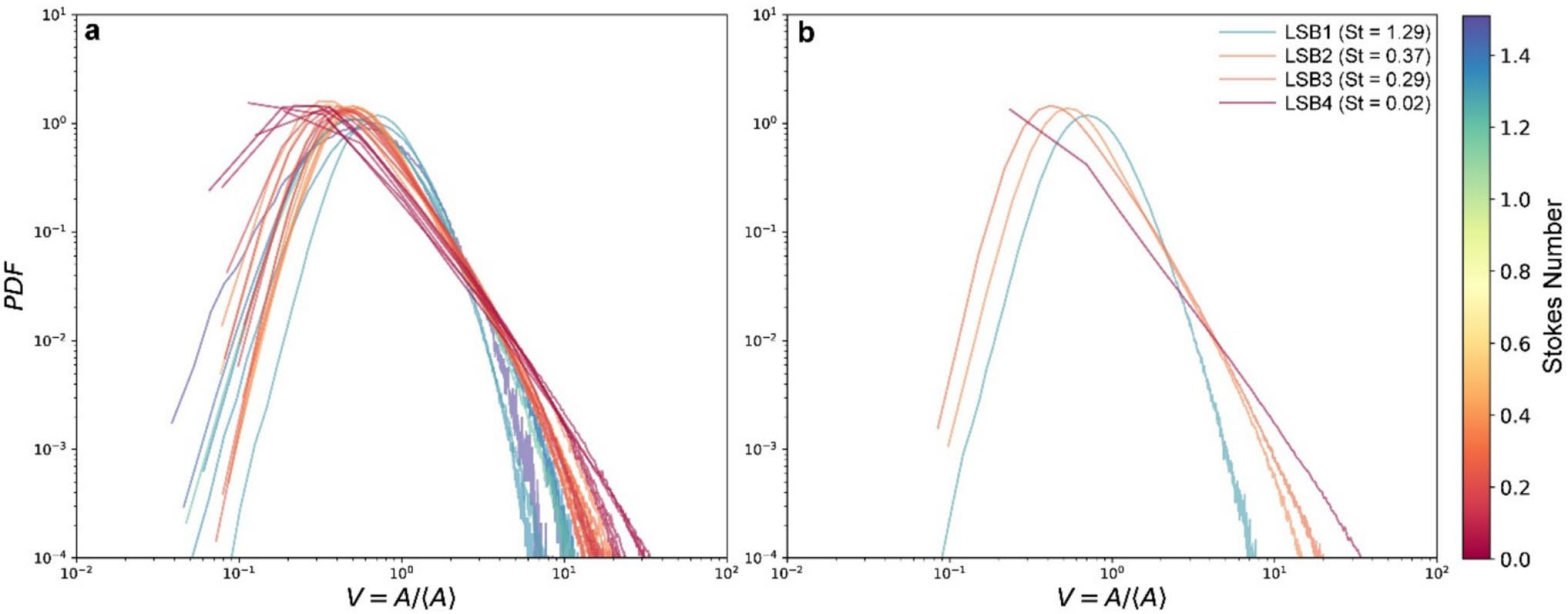
**a** Probability density function (PDF) of normalized Voronoi areas, *V*, for all experiments at different Stokes numbers (St). **b** PDFs for four selected experiments, each corresponding to a different PSD but with the same cone opening. The same colour bar for St applies to both panels. The comparison highlights the influence of particle size and release rate on particle distributions

To quantify the dynamics observed in the high-speed videos, we applied the previously outlined workflow and statistically analyzed the areas of the Voronoi cells. For each experiment, the particle coordinates were used to construct Voronoi diagrams at every time step. Then, the normalized cell areas were used to generate and compare the PDFs across different experiments to assess the spatial distribution of ash particles. Figure 4a shows the PDFs of the normalized Voronoi areas for all experiments, while Fig. 4b shows the PDFs for four selected experiments, each corresponding to a different PSD, for the same cone opening (which results in varying release rates depending on the particle size; Fig. 1e).

The evolution of the PDFs (Fig. 4a) reveals a clear trend with varying St. For larger particles (LSB1), the PDFs show a bell-shaped profile, peaking at larger normalized Voronoi areas, indicative of a more uniform particle distribution. This uniformity aligns with visual observations. As particle size decreases (from LSB2 to LSB4), the distributions become progressively narrower and more compact, with shorter left tails extending towards smaller normalized areas, while the right tails shift towards higher normalized areas. This trend mirrors the increasing tendency of smaller particles to cluster. The transition from well-defined, bell-shaped distributions for larger particles to compact, short-tailed distributions for smaller particles (Fig. 4b) highlights a significant shift in spatial distribution. A complementary comparison based on Voronoi-derived concentrations is provided in the Supplementary Material (Fig. S01), reproducing the same St-dependent trend.

**Fig. 5:**
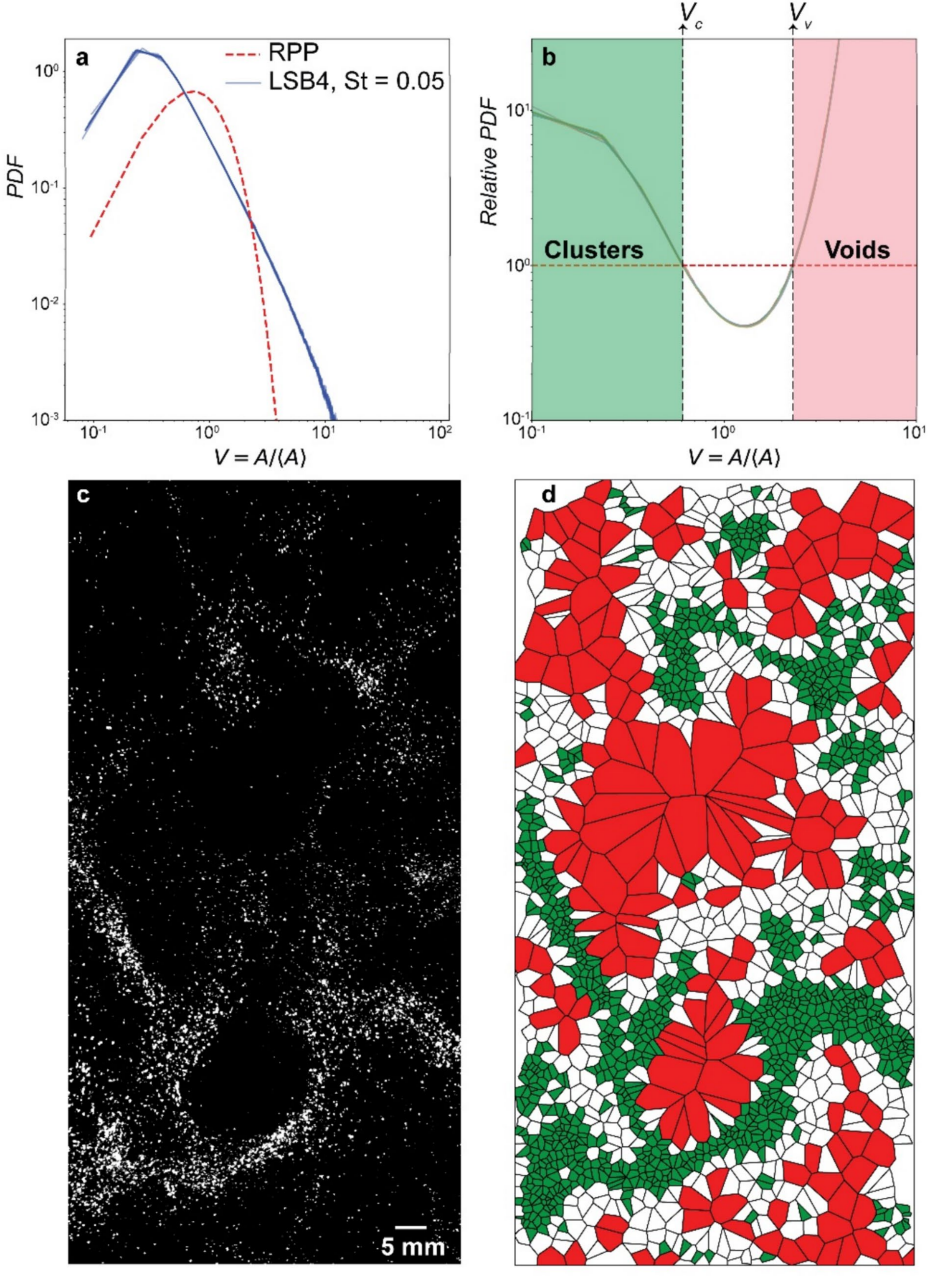
**a** PDFs of normalized Voronoi areas for 10 random subsets of the same experiment (solid blue lines, LSB4, St = 0.05), compared to an RPP (dashed red line). Each blue line represents the PDF calculated from a subset of 500 randomly selected frames. **b** Ratio of the experimental PDF to the RPP PDF. The intersections of these PDFs define the thresholds for clusters (*V*_*c*_) and voids (*V*_*v*_). **c** A representative raw frame from the experiment, and **d** Voronoi diagram corresponding to the frame in **c**, colour-coded to identify clusters (green), voids (red), and intermediate regions (white). Basic levels and contrast adjustments were applied to the frame in **c** for improving visibility

To further analyze the spatial distribution of particles, we compared the experimental PDFs to those of an RPP. The RPP, representing a purely random spatial distribution, serves as a reference to quantify deviations arising from the formation of regions of densely packed particles or particle-depleted regions. The experimental PDFs for all particle sizes deviate from the RPP. These deviations increase with a decrease in particle size, which exhibit a higher probability of both small and large normalized areas. For smaller PSDs, the normalized Voronoi–area PDF has heavier tails than the RPP: very large cells (*V* ≫ 1) are more frequent (more voids), and conversely, very small cells (*V* ≪ 1) are also more frequent (clusters; Fig. 5a). These deviations are characteristic of non-random particle distributions (e.g. Monchaux et al. 2010; Obligado et al. 2014). To quantify these regions for a given experiment, we determined two thresholds, *V*_*c*_ and *V*_*v*_, by locating the intersection points between the ratio of the experimental PDF and the RPP (Fig. 5b). These intersection points define two main regions: clusters, when *V* < *V*_*c*_, and voids, when *V* > *V*_*v*_ (Monchaux et al. 2010; Fig. 5b). These thresholds provide a quantitative framework for characterizing particle clustering and void formation, allowing for a detailed assessment of how particle size and release rates influence distribution patterns. Figure 5c and d visually illustrate our analysis by showing a RAW frame (Fig. 5c) and its corresponding Voronoi tessellation (Fig. 5d), color-coded based on *V*_*c*_ and *V*_*v*_. Such diagram highlights the regions identified as clusters (green), voids (red), and intermediate regions (white), confirming that smaller particles tend to form clusters surrounded by voids, as observed in the PDFs. It is important to note that these thresholds are unique to each experiment and show variability across the experimental dataset.

**Fig. 6:**
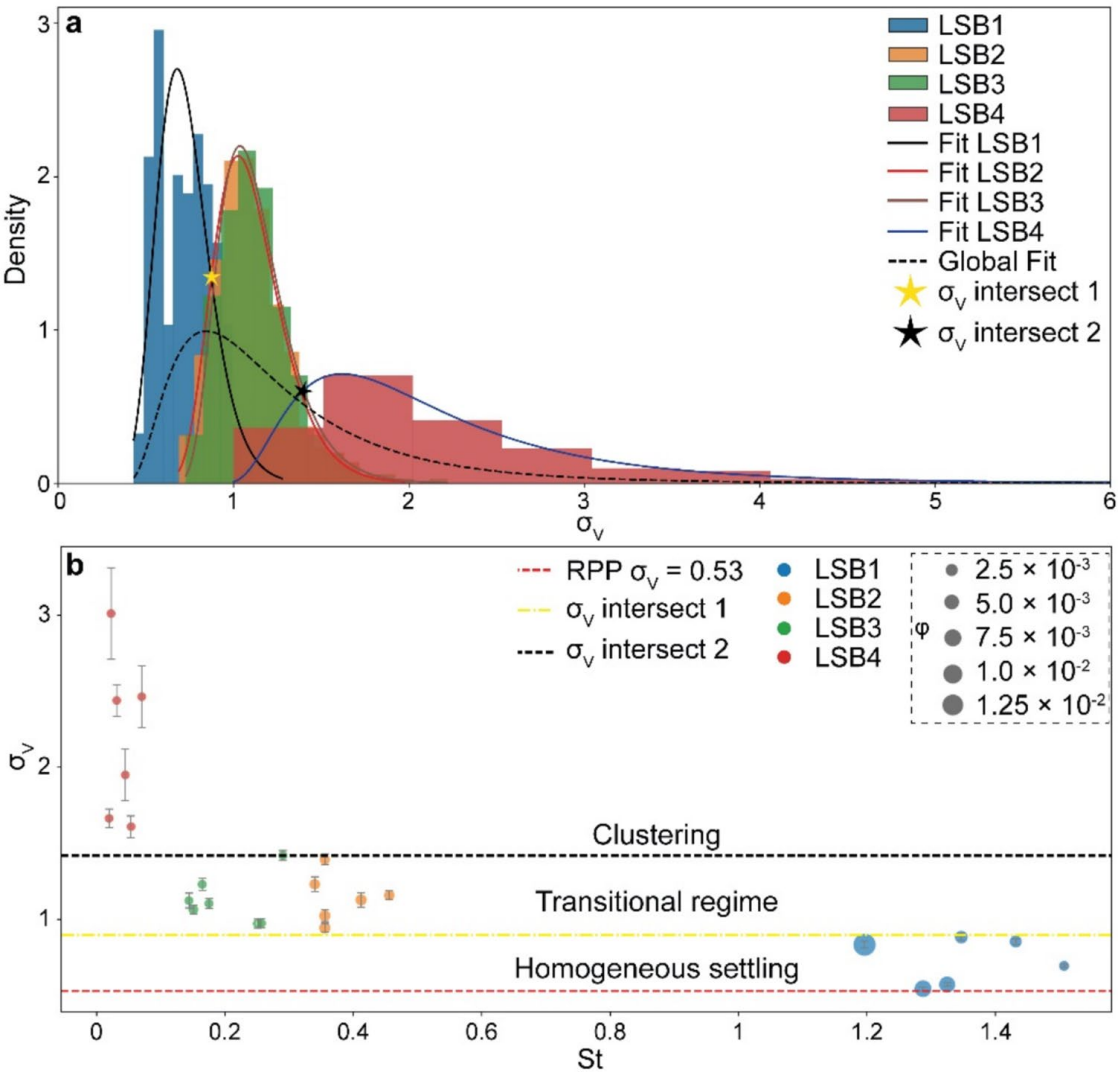
**a** Distributions of standard deviation of normalized Voronoi areas, *σ*_*V*_, for all particle size distributions (PSDs). For each experiment, we calculated *σ*_*V*_ over 100 subsets of 500 randomly sampled frames. Each distribution is individually fitted with log-normal distributions (solid lines). A global log-normal fit encompassing the entire *σ*_*V*_ dataset is also shown (dashed black line). Intersection points (*σ*_*V*_ intersect 1 and *σ*_*V*_ intersect 2) between the log-normal fits are indicated by the yellow and black stars, respectively. **b** Mean *σ*_*V*_ for each experiment as a function of Stokes number. Each *σ*_*V*_ value is an average calculated from 10 subsets of 500 randomly selected frames. The error bars in the plot represent the standard deviation of the *σ*_*V*_ values obtained from these 10 subsets, providing an estimate of the uncertainty associated with each mean value. Dashed yellow and black lines indicate the *σ*_*V*_ values for fits intersect 1 and 2, respectively. The red dashed line indicates the *σ*_*V*_ for an RPP (0.53). Symbol size in **b** represents the particle volume fraction, *φ*

Both the trends of PDFs and their comparison with an RPP suggest that particle size plays a crucial role in determining the distribution of ash particles within the falling column. Larger particles tend to promote more uniform settling, whereas smaller particles are more strongly influenced by flow dynamics, leading to increased clustering and more heterogeneous spatial distributions. The standard deviation of the Voronoi cell areas, *σ*_*V*_, serves as a robust proxy for quantifying the tendency of clustering observed in the PDFs, enabling a comparison of clustering across various experimental conditions. As already mentioned, for an RPP *σ*_*V*_ ≈ 0.53 (Monchaux et al. 2010); higher *σ*_*V*_ values therefore indicate a broader spread of our data points, reflecting stronger clustering and less uniformity relative to a random field. A lower standard deviation implies a more uniform distribution. Figure 6a summarizes *σ*_*V*_ for each PSD as density curves; each curve represents the variability of *σ*_*V*_ across 100 subsets of 500 RSF per experiment. The distributions for LSB1 and LSB4 are clearly distinct, while those for LSB2 and LSB3 overlap significantly. Overall, three main groups emerge, reflecting a clear trend (Fig. 6a): *σ*_*V*_ increases as particle size decreases, suggesting a stronger degree of clustering for smaller particles. The similarity between LSB2 and LSB3 suggests that these particle size ranges exhibit comparable susceptibility to fluid-particle interactions and similar clustering behaviours. All distributions are well described by log-normal fits. For LSB2 and LSB3, the fits overlap significantly, making their distributions statistically indistinguishable (Fig. 6a). The LSB1 and LSB2/3 fits intersect at *σ*_*V*_ ≈ 0.88, while the LSB2/3 fit intersects LSB4 at *σ*_*V*_ ≈ 1.42 (Fig. 6a; a similar analysis based on Voronoi-derived *φ* is provided in the Supplementary Material, Fig. S02a). These intersection points may represent critical transitions in the settling behaviour of the particles, suggesting a shift in the dominant mechanisms influencing their dynamics as a function of PSD.

Figure 6b shows the mean *σ*_*V*_ for each experiment, plotted as a function of St. Based on the fit intersections (Fig. 6a), we identified three distinct regions: a low-*σ*_*V*_ region (between the RPP *σ*_*V*_ of 0.53 and 0.88), an intermediate-*σ*_*V*_ region (between 0.88 and 1.42), and a high-*σ*_*V*_ region (above 1.42). LSB1 falls within the low-*σ*_*V*_ region, LSB2 and LSB3 fall both within the intermediate-*σ*_*V*_ region, and LSB4 falls within the high-*σ*_*V*_ region. By combining the above *σ*_*V*_ observations with the quantitative insights from the experimental PDFs and the qualitative visual observations of the settling dynamics, we can then associate each region with distinct settling behaviour: (1) *Homogeneous settling*: characterized by low *σ*_*V*_ values, this regime is primarily associated with LSB1 particles; (2) *transitional settling*: characterized by intermediate *σ*_*V*_ values, this regime encompasses both LSB2 and LSB3 particle types; and 3) *clustering regime*: characterized by high *σ*_*V*_ values, this regime is associated with LSB4 particles. Within each region, though, we observed variability in *σ*_*V*_ across different experiments for a given particle size distribution, suggesting that settling dynamics and clustering development within a specific size range are not only influenced by size, but also by release conditions and, consequently, by particle volume fraction, as also observed visually (Fig. 6b, Supplementary Videos 03–08; a similar analysis based on *σ*_*φ*_ as a function of St is provided in the Supplementary Material, Fig. S02b).

**Fig. 7:**
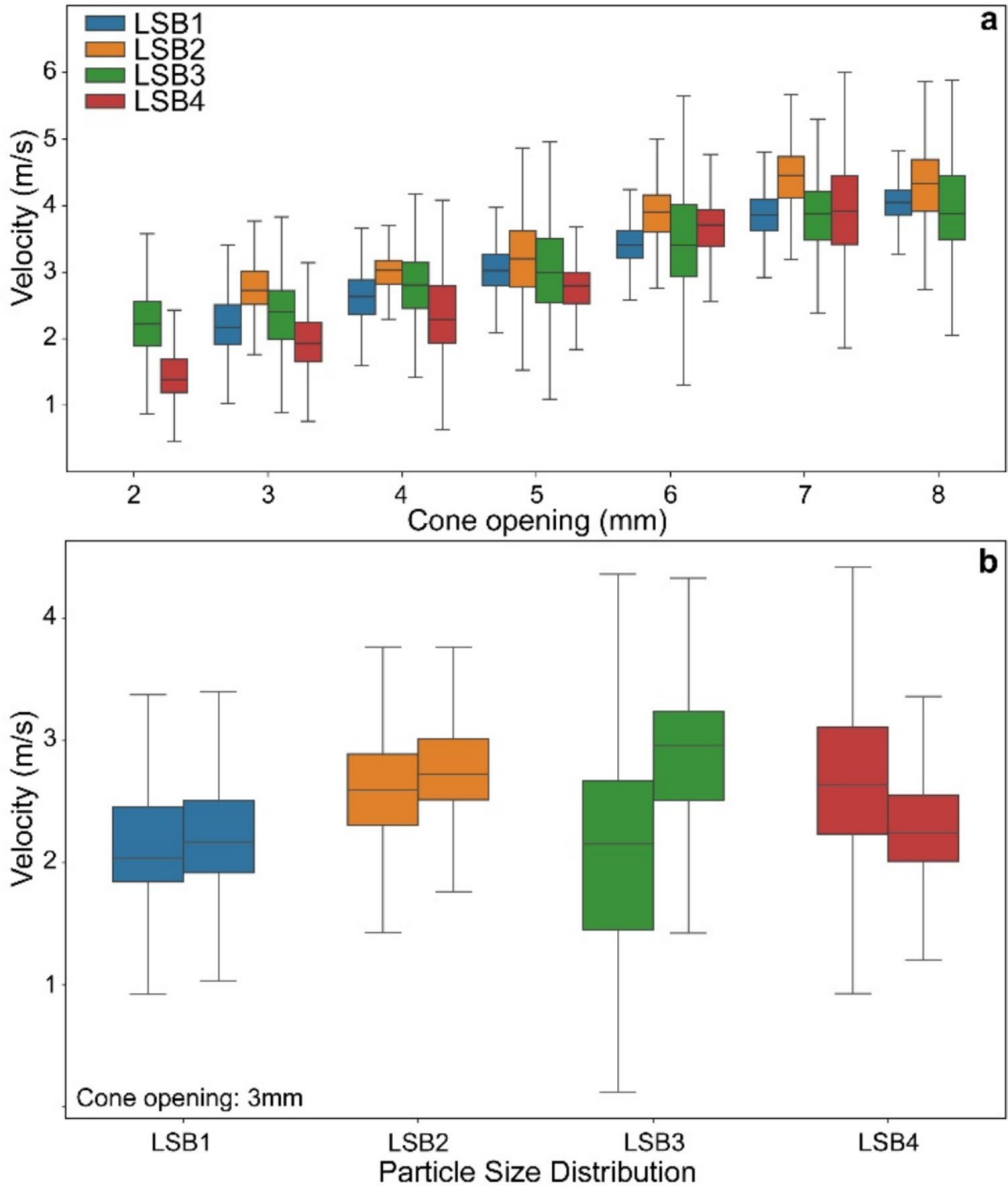
**a** Box plots showing the distribution of settling velocities, *u*_*p*,_ for each PSD at different release rates (i.e. increasing cone openings). **b** Box plots illustrating the variability of settling velocities within each PSD at different stages of the release process for a constant cone opening. The box plots represent the 25th and 75th percentiles, with the median indicated by a horizontal line. Whiskers extend to 1.5 times the interquartile range

Finally, we investigated the settling velocities of ash particles, *u*_*p*_, across different particle sizes and particle volume fractions within the column. Figure 7a illustrates the variation of *u*_*p*_ as a function of release rates for each PSD. Generally, for any PSD, increasing the release rate resulted in a corresponding increase in the overall *u*_*p*_, with the highest velocities observed at the highest release rates. Average *u*_*p*_ across all experiments ranged from approximately 1.1 m/s (for LSB4) to 4.5 m/s (for LSB2). There is a significant variability in settling velocities within each PSD (Fig. 7a). This trend was particularly pronounced for the smaller PSDs (LSB3 and LSB4). To further investigate this variability, we measured *u*_*p*_ at different stages of the release process for the same experimental conditions (Fig. 7b). LSB1 and LSB2 samples showed relatively low variability in velocities across different stages of the release, suggesting more uniform settling behaviour. In contrast, LSB3 and LSB4 were characterized by a significantly higher variability, with the highest *u*_*p*_ observed for particles settling within clusters and lower *u*_*p*_ associated with particles settling in particle-depleted regions (Fig. 7b).

## Discussions

Our experiments revealed significant insights into the clustering behaviour of settling volcanic ash particles. By creating repeatable and controlled ash columns, systematically varying PSDs and particle volume fractions, and imaging the process using PIV, we captured detailed data on particle motion and evolution of their distribution. Additionally, the application of Voronoi tessellation allowed us to quantify particle distributions, uncover spatial inhomogeneities, and gain deeper insights into clustering dynamics. While our experiments share similarities with studies on finger formation in density-stratified fluids (e.g. Manzella et al. 2015; Scollo et al. 2017; Fries et al. 2024), they diverge in focusing on particle behaviour within settling columns where turbulence and particle inertia—both influenced by particle size and volume fraction—dominate clustering development rather than buoyancy-driven instabilities.

**Fig. 8:**
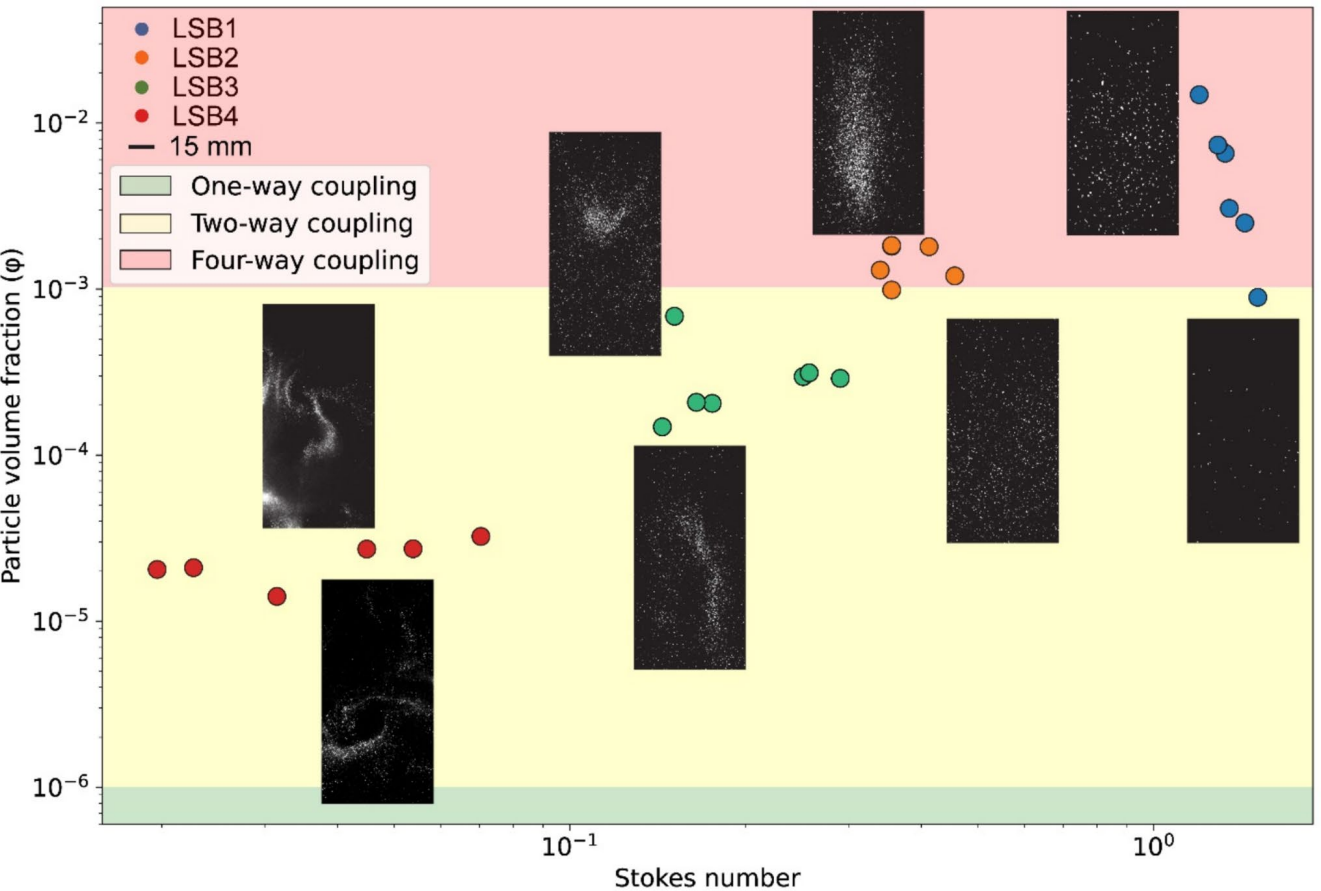
Different regimes characterizing particle transport in turbulence, defined by Stokes number (x axis) and particle volume fraction, *φ* (y axis), following definitions in Elghobashi (1994). Shaded regions mark coupling ranges based on *φ*: one-way (*φ* < 10^–6^), two-way (10^–6^ < *φ* < 10^–3^), and four-way (*φ *> 10^–3^) coupling. Symbols are coloured by PSD class (LSB1–LSB4). Representative frames are positioned at representative points showing the identified settling regimes for different PSDs and *φ*. Vortex-like preferential concentration appears at low St and low *φ* within the two-way range, whereas higher *φ* and St tend toward either more transitional or homogeneous settling. The same 15 mm scale bar applies to all images. Basic levels and contrast adjustments were applied to the frames for improving visibility

Gravitational instabilities in density-stratified systems were observed only for particles smaller than 125 μm (Scollo et al. 2017; Fries et al. 2024). Similarly, in our experiments, the strongest clustering is associated with LSB4 (< 125 μm), suggesting a critical size threshold below which clustering dynamics become dominant. Unlike stratified cases, however, clustering in our experiments persists across a wide range of concentrations. While fingers fail to develop below a critical concentration in stratified systems (Scollo et al. 2017; Fries et al. 2024), clustering in our columns remains modulated, not suppressed, by particle volume fraction. At low *φ*, particle–flow interactions enhance preferential concentration; at high *φ*, particle–particle interactions become more influential, leading to the formation of complex structures. Homogeneous settling is the exception, where clustering is minimal due to the dominant influence of particle inertia in larger particles, enabling them to overcome turbulent fluctuations, maintain a more consistent settling trajectory, and settle uniformly. Although LSB1 lies within *φ* ranges associated with four-way coupling (Fig. 8; Elghobashi 1994; Brandt and Coletti 2022), its large size (St > 1) produces strong inertial settling, with particles following near-ballistic paths and only rare, random collisions. On the contrary, medium-to-smaller particles (< 500 µm; St ≲1) are more responsive to turbulent eddies and show enhanced inhomogeneous distributions, promoting clustering. As particles fall, they induce flow, generating particle-driven convection and internal mixing. This entrainment is stronger for smaller particles and facilitates the development of segregated structures, with particle-rich and particle-poor regions coexisting. These particles (LSB2–LSB3) fall within the transitional regime, where the field organizes into large, elongated regions of both enhanced and depleted concentration, often oriented vertically and/or horizontally, rather than into compact, localized clusters (Fig. 8). The size of these structures increases with *φ*, consistent with intermittently coherent turbulence that amplifies segregation without producing fully discrete clusters (Fig. 8). This behaviour resembles the intermediate-*φ* regimes identified in large-scale pyroclastic density current experiments, where dense particle clusters embedded in turbulent structures enhance segregation and mobility without forming a single, continuous dense core (Breard et al. 2016; Breard and Lube 2017; Lube et al. 2020). Within this regime, LSB2 lies at the lower end of four-way coupling (*φ* ≳10^−3^) and, accordingly, shows more frequent collisions than LSB3; however, these events do not appear to be dominant enough to control the organization of the flow. LSB3, at lower *φ*, remains firmly two-way coupled (*φ* ≲10^−3^; Elghobashi 1994; Petersen et al. 2019; Brandt and Coletti 2022) with fewer collisions overall. In the clustering regime (LSB4), all experiments lie in two-way coupling (Fig. 8). Particles organize into compact, localized clusters, whose behaviour depends on loading. At lower *φ*, clusters are transient and ‘rotate’ with the flow, likely due to coupling with turbulent eddies (consistent with turbulence-particle coupling at St < 1). At higher *φ*, the collective downward motion of the particles drives a stronger downward air flux and a compensating return flow. This enhances drift into converging regions of the flow so that clusters become more gravity-driven, settling into well-defined vertically aligned structures dominated by particle inertia and collective settling dynamics (Fig. 8).

These concentration-dependent effects are also observable in the variations of *σ*_*V*_ for each PSD as a function of St (Fig. 6b). As expected, St < 1 particles follow the flow more closely, whereas St > 1 particles decouple and move more independently (e.g. Voth and Soldati 2017). However, in our experiments, the relationship between *σ*_*V*_ and St is not linear. The *σ*_*V*_ varies depending on the release rate and, consequently, *φ*, which influences settling velocity (Fig. 7) and, thus, the effective St. This highlights the complex role of *φ* in modulating the scale and intensity of particle segregation and clustering within the regimes.

These findings demonstrate that particle clustering might not be solely confined to the finer ash size distributions (< 125 µm), as suggested for the development of gravitational instabilities (e.g. Scollo et al. 2017). We observed distinct levels of clustering across a range of particle sizes between 500 and 63 µm, thus including larger particles and different values of *φ*. This St–*φ* dependence is consistent with multiphase models of pyroclastic density currents in which St–controlled segregation and clustering partition the flow into a dense, near-surface core and an overlying, more dilute region (Burgisser and Bergantz 2002) and with experimental and theoretical work highlighting an intermediate-*φ* regime where particle–gas interaction and cluster-induced turbulence are particularly effective (Lube et al. 2020). In our experimental columns, turbulent dynamics (particle–flow coupling) and particle–particle interactions both contribute to cluster formation, with their relative importance shifting with *φ*. Clustering is most pronounced for low–intermediate St (≈0.02–0.3) within two-way coupling and *φ* between ~ 10^–5^ and 10^–3^. This range reflects our choice of a large-scale fluid timescale when defining St; in contrast, for particles in water or droplets in air, the commonly reported condition of maximum clustering is at St ≈ 1, as it is based on a Kolmogorov-scale Stokes number (e.g. Brandt and Coletti 2022; Voth and Soldati 2017). Using a shorter Kolmogorov timescale, or equivalently a smaller characteristic length such as the column diameter or the size of the largest eddies, would shift all of our St values upward by a factor of a few, placing the runs with strongest clustering into the canonical St ≈ 0.1–1 band without changing their ordering in St–*φ* space. Furthermore, this result is not sensitive to grain-size-dependent density variations: the density of Laacher See ash is nearly size-independent over the PSDs considered (Douillet et al. 2014), so any density correction would only mildly rescale St and would not move PSDs between regimes. Clustering weakens as St approaches/exceeds 1 and also as *φ* increases toward the four-way coupling. Increasing *φ* within two-way coupling tends to make clusters more coherent (gravity-aligned packets; LSB3). This suggests that preferential concentration may develop not only at the boundaries of density layers but also within the bulk of the settling volcanic cloud, where both low and high concentrations coexist and turbulence persists. The occurrence and intensity of clustering depend on a combination of factors, including particle size, volume fraction, and turbulence levels; more generally, they peak when the particle response time is comparable to the eddies that organize the flow, consistent with inertial-particle theory and established coupling arguments (e.g. Elghobashi 1994; Petersen et al. 2019). In a volcanic environment, these regimes encompass both drag-induced and wake-induced clustering of polydisperse particles, as described by Lube et al. (2020). Our experiments sample lower *φ* than typical PDC studies: clustering is most pronounced for the finer PSDs (LSB3–4) at *φ* ≈ 10^–5^–10^–3^, whereas the coarsest, highest-*φ* runs (LSB1, *φ* ≳10^–2^) are dominated by nearly homogeneous, gravitational settling. This has important implications for VATDMs and models of ash aggregation. Analogous cluster focusing and turbulent particle–gas interactions have been linked to enhanced dynamic pressures and spatially variable impact in dilute pyroclastic density currents (Brosch et al. 2022; Uhle et al. 2024), suggesting that the spatial organization of *φ* can strongly modulate volcanic hazards. Clustering introduces significant spatial variability that is often neglected in VATDMs, resulting in inaccurate forecasts (Costa et al. 2013; Buckland et al. 2022). Furthermore, current aggregation models for VATDMs frequently assume that aggregation occurs in regions of maximal mean concentration, where particle–particle interactions are more frequent. Yet clustering can shift collision regions away from those maxima by locally increasing number density and relative velocities. Accurately representing the spatial organization of *φ* is therefore critical. Neglecting clustering may lead to misplacing and miscalculating aggregation efficiency. Therefore, incorporating preferential concentration, especially where particle size, turbulence intensity, and concentration align to promote clustering, improves forecasts of cloud evolution, dispersion, and fallout.

The effect of clustering on settling velocities is also significant. Del Bello et al. (2017) demonstrated that inter-particle collisions within a particle flow and increasing concentrations can increase *u*_*p*_ of ash within falling columns. Similarly, our experiments show that higher particle concentrations lead to increased *u*_*p*_ across all particle size distributions (Fig. 7a). Figure 7a also reveals significant variability in settling velocities within each PSD. This variability can be attributed to several factors, including the wide size ranges for each PSD, where larger particles within a given range generally exhibit higher *u*_*p*_. Additionally, particle aggregation may occur, as aggregates settle faster than individual particles (Lane et al. 1993). Moreover, particle-driven convection and the development of clusters can produce differential settling velocities within the column. Del Bello et al. (2017) reported, for LSB samples in the 1–0.5 mm range (equivalent to our LSB1), *u*_*p*_ between 1 and 6 m/s, with modal values around 2.5–3 m/s. Our LSB1 samples were characterized by similar *u*_*p*_ ranges (1–7 m/s), with average values of 2.2 and 4 m/s for the lowest and highest release rates, respectively. For LSB2–4, velocities ranged from 0.4 to 6 m/s, with average values between 1.1 and 4.5 m/s, increasing with higher release rates (Fig. 7; Table 1). However, this relationship is not always straightforward. At higher release rates, LSB2 and LSB3 often show higher median velocities than LSB1 or LSB4, despite the latter being characterized by the largest particles. This suggests that clustering, which is more pronounced for LSB2 and LSB3 at higher release rates (Fig. 8), plays a significant role in enhancing *u*_*p*_. Within clusters, particles may experience reduced drag due to shielding effects, where outer particles deflect airflow and reduce resistance for inner particles, allowing the entire structure to settle more rapidly. This type of cluster-enhanced settling is consistent with large-scale multiphase experiments and simulations of pyroclastic density currents, where particle clusters fall several times faster than individual particles of equivalent size and generate cluster-induced turbulence in their wakes (Lube et al. 2020). For reference, still-air terminal velocities estimated for representative particle sizes are of the order of 3.5, 1.9, 0.8, and 0.3 m/s for LSB1–4, respectively; at the highest release rates, median velocities in LSB2–3 reach ≈4.5 and 4 m/s, i.e. up to about five times the single-particle value. In contrast, dispersed particles encounter higher individual drag forces, slowing their fall. This collective motion of clustered particles effectively disrupts the typical correlation between particle size and settling velocity, as smaller, clustered particles can achieve higher settling velocities. Such dynamics might significantly influence the evolution of the ash cloud, potentially accelerating deposition rates of finer and unaggregated particles and altering the spatial distribution of ashfall. This has important implications for remote sensing techniques that use particle velocity as a proxy for size. Instruments such as Doppler radars often assume a direct correlation between particle size and settling velocity, with larger particles and aggregates expected to fall faster. However, clustering challenges this assumption, particularly for intermediate-sized particles like LSB2 and LSB3, which exhibit enhanced settling velocities. This can lead to overestimations of particle size when velocity measurements are interpreted without accounting for clustering effects. Furthermore, many models assume that settling velocities directly correlate with particle size, often neglecting the influence of clustering (e.g. Folch 2012; Beckett et al. 2022). Incorporating clustering dynamics into these models is essential for improving estimates of aggregated particle size distributions and accurately simulating ash transport and deposition processes. Finally, understanding clustering could help differentiate between true aggregates and clusters of unbound particles, offering valuable insights into the mechanics of ash transport and the spatial variability of deposition.

## Final remarks

Our findings underscore the crucial role of particle clustering throughout the volume of the dispersing ash cloud in influencing its transport and deposition. While our study provides valuable insights into the fundamental mechanisms driving particle clustering at the millimetres scale within a limited and controlled experimental setting, further research is necessary to fully understand its implications for larger-scale volcanic clouds. The influence of larger-scale turbulent structures and atmospheric conditions on particle clustering dynamics needs to become an area of active investigation. Towards this goal, the novel experimental methods and the application of Voronoi tessellation presented in our study offer a new perspective on how particle size, volume fraction, and turbulent dynamics govern clustering within volcanic ash settling columns. These temporal fluctuations, driven by the above parameters and evolving flow structures, highlight the variability in the spatial distribution of particles, underscoring the dynamic nature of clustering and dispersion within the column. By integrating our experimental methods with Voronoi tessellation, we not only provide new insights into clustering dynamics but also establish a robust framework for understanding the role of turbulence and concentration effects on particle behaviour at a fine scale. This marks a significant advancement in both experimental design and data analysis for studying the settling dynamics of volcanic ash particles.

## Supplementary information

Below is the link to the electronic supplementary material.

ESM 1 (PDF 829 KB)

ESM 2 (AVI 159 MB)

ESM 3 (AVI 188 MB)

ESM 4 (AVI 212 MB)

ESM 5 (AVI 269 MB)

ESM 6 (AVI 259 MB)

ESM 7 (AVI 241 MB)

ESM 8 (AVI 229 MB)

ESM 9 (AVI 222 MB)

## Data Availability

Example datasets supporting this study are provided as supplementary material. Additional data generated and analyzed during the study are available from the corresponding author upon reasonable request.
