# Supplementary material for: Voronoi-based analysis of clustering dynamics in experimental volcanic ash clouds
Source: Bull Volcanol. 2026 Jan 21;88(2):15. doi: 10.1007/s00445-025-01933-x (PMC12823729; doi:10.1007/s00445-025-01933-x)
Supplement: Supplementary file 1 — (PDF 829 KB) [file 445_2025_1933_MOESM1_ESM.pdf]

Voronoi-Based Analysis of Clustering Dynamics in Experimental Volcanic Ash Clouds

Antonio Capponi, Corrado Cimarelli, Pablo Mininni

Corresponding author: antonio.capponi@min.uni-muenchen.de

Supplementary Material

The list of supplementary figures and files is provided below with a description of their type and content.

Supplementary Figures:

Figure S01 shows a comparison of the PDFs of particle concentrations for the same curves as Fig. 4 in the main text. The concentrations were obtained from the Voronoi areas as $C = 1/A$, as the area of each Voronoi cell corresponds to the specific area of each particle, and thus its inverse is the particle density. The concentration is normalized by the mean concentration in all panels. Figure S02 shows the same analysis as in Fig. 6 but based on the particle volume fraction φ, again derived from the Voronoi tessellation data.

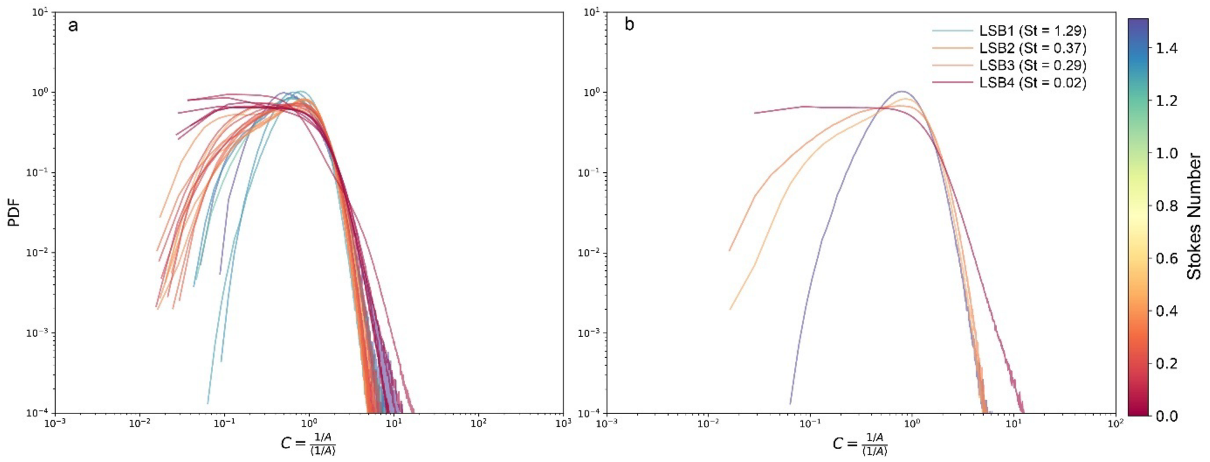

Fig. S01 (a) Probability Density Function (PDF) of Voronoi-derived number concentration from 2D tessellations. For each particle, concentration is computed as C = 1/A, where A is the Voronoi cell area, and then mean-normalized per experiment as C = C/⟨C⟩ = (1/A)/⟨1/A⟩. PDFs are estimated from 5000 randomly sampled frames per experiment and normalized to unit area. (b) PDFs for four selected experiments, each corresponding to a different PSD but with the same cone opening. The same colour bar for St applies to both panels. As in Fig. 4 in the main text (area-based PDFs), larger particles (higher St) show broader, more bell-shaped distributions centred near C ≈ 1, whereas smaller particles shift toward higher C and develop heavier right tails, indicating stronger clustering.

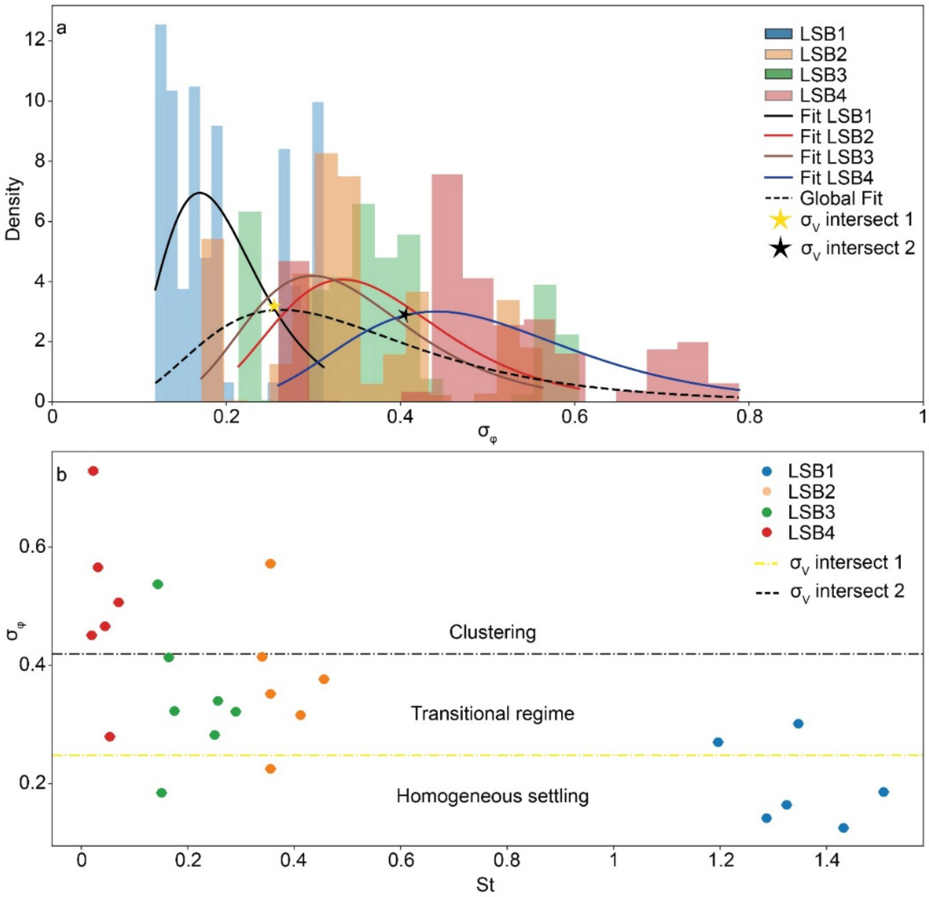

Fig. S02 (a) Distributions of the standard deviation of the frame-averaged particle volume fraction (σ_φ) derived from the Voronoi tessellation. For each PSD, histograms show the variability of σ_φ across 100 subsets of 500 randomly sampled frames per experiment; solid curves are log-normal fits and stars mark the separation thresholds defined as the midpoints between the intersections of adjacent PSD fits. (b) Mean σ_φ for each experiment versus Stokes number; horizontal lines indicate the two intersection values from panel (a), used as visual guides for the Homogeneous, Transitional, and Clustering regimes. Compared with the area-based analysis (σ_V) shown in Fig. 6, the φ-based trends appear less sharply separated because φ introduces additional scalings (depth of field and particle volume) and, through volume weighting, is more sensitive to polydispersity and experiment-to-experiment variability.

Supplementary Videos:

- *Supplementary Video 01, avi*: Example of a high-speed recording (5000 frames per second) of an experimental run using Laacher See ash samples LSB1, 1000–500 µm. The experiment was conducted with a 3 mm cone opening, resulting in a release rate of ~8.25 g/s and an estimated concentration of 8.04×10^1 g/m³ (calculated assuming a constant column diameter and using the average settling velocity). The actual duration of the video is 0.3 s, but it has been saved at 48 fps for playback

- *Supplementary Video 02, avi*: Example of a high-speed recording (5000 frames per second) of an experimental run using Laacher See ash samples LSB1, 1000–500 µm. The experiment was conducted with a 6 mm cone opening, resulting in a release rate of ~56 g/s and an estimated concentration of 3.68×10^2 g/m³ (calculated assuming a constant column diameter and using the average settling velocity). The actual duration of the video is 0.3 s, but it has been saved at 48 fps for playback

- *Supplementary Video 03, avi*: Example of a high-speed recording (5000 frames per second) of an experimental run using Laacher See ash samples LSB2, 500–250 µm. The experiment was conducted with a 3 mm cone opening, resulting in a release rate of ~24 g/s and an estimated concentration of 2.03×10^2 g/m³ (calculated assuming a constant column diameter and using the average settling velocity). The actual duration of the video is 0.3 s, but it has been saved at 48 fps for playback

- *Supplementary Video 04, avi*: Example of a high-speed recording (5000 frames per second) of an experimental run using Laacher See ash samples LSB2, 500–250 µm. The experiment was conducted with a 6 mm cone opening, resulting in a release rate of ~70 g/s and an estimated concentration of 4×10^2 g/m³ (calculated assuming a constant column diameter and using the average settling velocity). The actual duration of the video is 0.3 s, but it has been saved at 48 fps for playback

- *Supplementary Video 05, avi*: Example of a high-speed recording (5000 frames per second) of an experimental run using Laacher See ash samples LSB3, 300–125 µm. The experiment was conducted with a 3 mm cone opening, resulting in a release rate of ~20 g/s and an estimated concentration of 1.84×10^2 g/m³ (calculated assuming a constant column diameter and using the average settling velocity). The actual duration of the video is 0.3 s, but it has been saved at 48 fps for playback

- *Supplementary Video 06, avi*: Example of a high-speed recording (5000 frames per second) of an experimental run using Laacher See ash samples LSB3, 300–125 µm. The experiment was conducted with a 6 mm cone opening, resulting in a release rate of ~81 g/s and an estimated concentration of 4.92×10^2 g/m³ (calculated assuming a constant column diameter and using the average settling velocity). The actual duration of the video is 0.3 s, but it has been saved at 48 fps for playback

- *Supplementary Video 07, avi*: Example of a high-speed recording (5000 frames per second) of an experimental run using Laacher See ash samples LSB4, 125–63 µm. The experiment was conducted with a 3 mm cone opening, resulting in a release rate of ~21 g/s and an estimated concentration of 2.37×10^2 g/m³ (calculated assuming a constant column diameter and using the average settling velocity). The actual duration of the video is 0.3 s, but it has been saved at 48 fps for playback

- *Supplementary Video 08, avi*: Example of a high-speed recording (5000 frames per second) of an experimental run using Laacher See ash samples LSB4, 125–63 µm. The experiment was conducted with a 6 mm cone opening, resulting in a release rate of ~66 g/s and an estimated concentration of 4.12×10^2 g/m³ (calculated assuming a constant column diameter and using the average settling velocity). The actual duration of the video is 0.3 s, but it has been saved at 48 fps for playback
